# Role of nerve growth factor and its TRKA receptor in normal ovarian and epithelial ovarian cancer angiogenesis

**DOI:** 10.1186/s13048-014-0082-6

**Published:** 2014-08-10

**Authors:** Carolina Vera, Verónica Tapia, Margarita Vega, Carmen Romero

**Affiliations:** Laboratorio de Endocrinología y Biología Reproductiva, Hospital Clínico Universidad de Chile, Santos Dumont # 999, Santiago, Chile; Departamento de Obstetricia y Ginecología, Facultad de Medicina, Universidad de Chile, Santiago, Chile; Advanced Center for Chronic Diseases (ACCDiS), Santiago, Chile

**Keywords:** Angiogenesis, Vascular endothelial growth factor, Nerve growth factor, TRKA, Normal ovary, Epithelial ovarian cancer

## Abstract

In normal ovarian function a controlled angiogenesis is essential. Several growth factors are involved in this process, such as the vascular endothelial growth factor (VEGF) and nerve growth factor (NGF). The angiogenesis process in the normal ovary is a tightly controlled process that occurs in each ovarian cycle. Also, angiogenesis is critical for ovarian cancer development and it is responsible for tumor spread, metastasis and its peritoneal dissemination. Ovarian cancer is the fifth leading cause of cancer death in women and it is distinguished as the most lethal gynecologic cancer. In recent years angiogenesis has been given considerable attention in order to identify targets for developing effective anti-tumor therapies. Several molecules have been reported to promote angiogenesis, such as platelet-derived growth factor (PDGF) and its receptors, the angiopoietin/Tie ligand/receptor system and fibroblast growth factor (FGF). Primarily, VEGF has been identified to play key roles in driving angiogenesis. The above-mentioned molecules are candidate drug targets. Used in combination with other treatments, anti-angiogenic therapies have managed to reduce disease progression. The present review is focused in NGF and its high affinity receptor tyrosine kinase A (TRKA). The expression of VEGF, proliferation and the angiogenesis process in ovarian cancer is importantly induced by NGF, among other molecules.

## Introduction

The ovary is one of the few adult organs that undergo angiogenesis at regular intervals. In ovarian cancer, disturbances in the angiogenic process are a key factor in its development and subsequent progression [1]. Ovarian cancer is the most lethal gynecological cancer; it remains with a low survival rate [2], and therefore, understanding the mechanisms governing angiogenesis is important in order to develop new strategies for its treatment. Neurotrophins have been shown to be involved in both normal ovarian and ovarian cancer angiogenesis [3,4]; thus, targeting their signaling signifies a novel therapeutic opportunity.

Neurotrophins and their receptors are involved in mammalian ovarian development [5,6]. Besides, in the normal human ovary, the neurotrophin nerve growth factor (NGF) and its high affinity tyrosine kinase receptor (TRKA) are expressed in granulosa cells [7], and through the activation of TRKA, NGF induces an increase in the vascular endothelial growth factor (VEGF) expression [8]. In human epithelial ovarian cancer (EOC) cells, NGF and TRKA are also expressed [8], and the active form of TRKA has been associated with poor patient outcome [9]. This review will focus on our research efforts regarding the connection between NGF, TRKA and VEGF, and how these molecules have a relevant role in ovarian cancer progression. We will discuss how NGF and TRKA are involved in angiogenesis, cell proliferation and progression of EOC.

### NGF and TRKA in the normal ovary

That neurotrophin or neuropeptide family and particularly NGF are critical for cell survival and differentiation in both the central and peripheral nervous system [10,11] and their important role for non-neuronal tissue development [12,13] are known facts. Such functions were suggested by studies that evidenced the presence of high affinity neurotrophin receptors in a variety of non-neuronal tissues, including the cardiovascular, endocrine, immune and reproductive systems [14].

Although all the functions developed by neurotrophins in the ovary have not been identified yet, to date it is clear that neurotrophins represent the trophic support for the sympathetic innervation of the organ and that they play a significant role during ovarian development periods that are key to the reproductive function: early follicular development [5] and ovulation [6]. Neurotrophins facilitate early follicular development during the two sequential phases of such process, which are: differentiation of primordial follicles towards primary follicles and growth of the latter into secondary follicles [5]. Such stages are apparently related to the ability of nerve growth factor (NGF) to act on the proliferation of both granulosa cells as well as theca cells [5], and to follicular stimulating hormone (FSH) receptor synthesis in granulosa cells [15]. Upon the first ovulation, NGF contributes to the ovulatory cascade by increasing the release of E2 prostaglandins (PGE2) [6], decreasing gap-junction communication and inducing theca cell proliferation in pre-ovulatory follicles [16].

All the processes described above have been demonstrated in rodents. However, there is evidence showing that neurotrophins and their receptors are also present in the fetal human ovary [17] and that human granulosa cells secrete another neuropeptide such as Brain-Derived Neurotrophic Factor (BDNF) that might be participating in oocyte maturation regulation [18]. Results obtained by our group have shown that in the normal human ovary, NGF and its high affinity TRKA receptor are mainly expressed in granulosa cells of antral and preantral follicles, as well as in theca cells of antral follicles. In addition to such findings, we also observed that NGF increases both the expression of FSH receptors, as well as estradiol secretion by cultured human granulosa cells [7].

The expression of NGF and its receptor on human granulosa cells of preantral and antral follicles [7] matches the expression of VEGF [19]. The latter suggests that at least some ovarian cells are prepared to provide the paracrine stimulus required for new blood vessel growth. During reproductive life, VEGF participates in the cyclic growth of ovarian follicles as well as in the development and maintenance of the corpus luteum. VEGF expression and secretion are induced via the activation of both FSH as well as luteinizing hormone (LH) receptors. VEGF expression and production within the ovary are critical to the normal reproductive function. Angiogenesis defects may contribute to a variety of disorders including anovulation, infertility, miscarriage, ovarian hyperstimulation syndrome and ovarian cancer [20].

Because TRKA receptor expression in the ovary takes place before ovulation [6] similarly to what happens with VEGF [20], it might be presumed that both factors could be acting concomitantly in important processes of ovarian function and possibly in angiogenic processes that take place during the follicular and luteal phases of the ovarian cycle. Additionally, NGF [7], as well as VEGF [20] is also reputed to be involved in certain ovarian pathologies such as polycystic ovary.

When ovaries are autotransplanted to an ectopic site in the rat, they swiftly recover functionality [21]. After 48 hours of ovary transplantation, they exhibit a massive revascularization together with an increased VEGF expression through a gonadotropin-related process [22]; four days after transplantation they recover the ability of performing a negative feedback on the hypothalamus-pituitary axis [21].

Therefore, NGF alone or in combination with other biologically active endogenous molecules, is able to exert its action on endothelial cells and more probably on the angiogenic activity [23]. It has also been suggested that NGF stimulates VEGF production by peripheral sensory neurons [23].

An increase in blood vessel density was observed during the neovascularization process of the superior cervical ganglion in newborn rats, when such animals were treated with NGF. This effect is directly correlated with an increased VEGF expression [24]. Such results suggest that angiogenesis may be indirectly regulated through NGF.

For such reason, knowing that human granulosa cells express NGF and their high affinity receptor (TRKA), we were interested in studying whether NGF was able to regulate VEGF expression in cultures of such cells. Our findings showed that in fact, NGF increases VEGF expression in an autocrine fashion, by activating TRKA receptor and through the subsequent activation of the MAPK – ERK and PI3K and AKT signaling cascade [25].

The results obtained by our work group suggest that in the adult human ovary NGF might be involved in normal ovarian angiogenesis, through VEGF expression and FSH receptor expression and also through estradiol secretion prior to ovulation [7,25].

### Ovarian cancer

Ovarian cancer is detected in late stages because it is a silent pathological condition, with poor therapeutic response and thus with a high mortality rate. Survival rates do not exceed 31% of patients diagnosed with ovarian cancer [2]. Ovarian carcinoma pathogenesis is still unclear. Many mechanisms have been proposed to explain ovarian cancer etiology. The Fathalla Theory suggests that repeated ovulations represent a trauma to the ovarian epithelium that renders such cells more susceptible to malignant transformation [26]. Classically, EOC has been described as originating from the epithelium (OSE). The OSE is a single layer of modified mesothelial cells that overlays the ovarian surface, and is separated from ovarian stoma by the basal lamina. Inclusion cysts (present within the ovarian stroma and lined by the OSE cells) and ovarian surface invaginations (lined by OSE cells) have been described as preferred sites for neoplastic transformation [27]. This might be explained by the fact that the ovary undergoes an injury with each ovulation and thus, by virtue of local factors, the epithelium begins uncontrolled proliferation [28]. The latter is supported by evidences demonstrating that EOC originates through the clonal expansion of one single transformed stem cell [29]. However, recent studies have suggested that in some cases, ovarian cancer might originate from extraovarian epithelial lesions that settle on the ovary and originate neoplasia [30]. Nevertheless, there are no conclusive evidences demonstrating or supporting such theory.

The series of events involved in the genesis, progression and metastasis of EOC has not been yet established. Evidence suggests that tumor progression of EOC occurs slowly from benign or borderline tumors, or may develop rapidly de novo from the OSE or inclusion cysts [29]. The majorities of the malignant ovarian tumors are epithelial (80%) and are highly angiogenic [27]. Angiogenesis is a prerequisite for solid tumor growth after a short avascular phase [31]; it involves capillary endothelial cell proliferation [32] as well as migration [33]. Endothelial cells in human tumors have a proliferation rate that is 50 or 200 fold higher than endothelial cells in normal adult endothelial tissue [32].

The angiogenic process has a theoretical significance in the context of ovarian cancer for two reasons. First, the angiogenesis process occurs in a very controlled fashion as part of the normal ovarian function, during ovulation [20]. The latter suggests that at least some ovarian cells are ready to provide the paracrine stimulus required for the growth of new blood vessels, and that, upon transformation, such ability is present early during tumor development. Second, the large size that characterizes ovarian tumors requires an angiogenesis process for tumor sustenance [31].

VEGF is an angiogenic factor and potent mitogen for the vascular endothelium and one of the most important factors in ovarian angiogenesis [34]. Besides VEGF, other molecules have been identified to play important roles in promoting and maintaining angiogenesis during the oncogenic process. Such molecules include the platelet-derived growth factor (PDGF) and its receptors, the angiopoietin/Tie ligand/receptor system and the fibroblast growth factor (FGF) [35]. PDGF leads to upregulation of angiogenic events and to tumor growth through the activation of its receptor, PDGFR. It acts together with VEGF to induce vessel formation and to stabilize the newly formed vessels. PDGF has been found to increase in human ovarian cancer tumor cells compared to normal ovarian epithelium [35]. Angiopoietin, on the other hand, has two isoforms, Ang1 and Ang2, both of which can interact with the Tie 2 receptor to induce new vessel production. In cancer, Ang2 has also been linked to metastasis [36]. Finally, FGF is another molecule that plays a role in ovarian carcinogenesis. It can induce tumor cell proliferation and it plays a role in promoting angiogenesis in collaboration with VEGF. It may also be secreted into malignant ascites promoting both angiogenesis and cancer progression. Its receptor, FGFR, is able to interact with adhesion molecules, promoting metastasis [37]. Importantly, both PDGF and FGF levels can be correlated with prognosis [35].

Currently, ovarian cancer treatment consists on the surgical removal of the affected ovary or even a bilateral oophorectomy, followed by chemotherapy. Most patients respond to this line of treatment, however, the majority of patients recur and the tumor develops drug resistance [38]. In this context, given the low overall survival for ovarian cancer, there is a great need for new therapeutic approaches that are able to give more durable responses.

Angiogenesis appears as one of the main focuses for future drug development. Molecular-targeted therapy, in combination with cytotoxic treatment, may prove to be a good strategy to improve ovarian cancer prognosis. Up to the present time, there are no FDA-approved anti-angiogenic drugs used in ovarian cancer treatment, however, several clinical trials against the VEGF, Ang, FGF and PDGF pathways have managed to reduce disease progression [39].

### NGF and TRKA in EOC

In addition to the importance of angiogenesis in tumor processes, cancerous cells are characterized by a lack of cell growth regulation control. This occurs in part through signaling generated from a variety of growth factor receptors, such as tyrosine kinase receptors [40,41]. In many cell types, overexpression of neurotrophin receptors or changes in the activity of intracellular signal transduction cascades are involved in malignant transformation [40].

Some authors suggest that the interaction between NGF and its specific receptor TRKA might be involved in the growth of some non-neuronal cancers. Likewise, an overexpression of neurotrophin receptor has been demonstrated in cancers from various tissues such as: thyroid [40,41], lung [42], esophagus [43], prostate [44], breast [45] and also in ovarian cancer [46] where expression of TRKA receptors was detected in 82% of solid epithelial tumors [32]. Neurotrophin receptor expression might have a more than prognostic biological relevance in ovarian carcinoma [46]; however, the few studies regarding the participation of neurotrophins and their receptors in such cancer do not enable reaching a valid conclusion. For such reason it was important to assess cell proliferation in EOC and some molecules involved in NGF- mediated TRKA activation signaling pathway. Such study revealed an increase in p-AKT protein levels, in BCL2/BAX ratio, c-myc and Ki67. In turn, levels of FOXL2, a transcription factor involved in apoptosis, were decreased [47].

**Figure 1:**
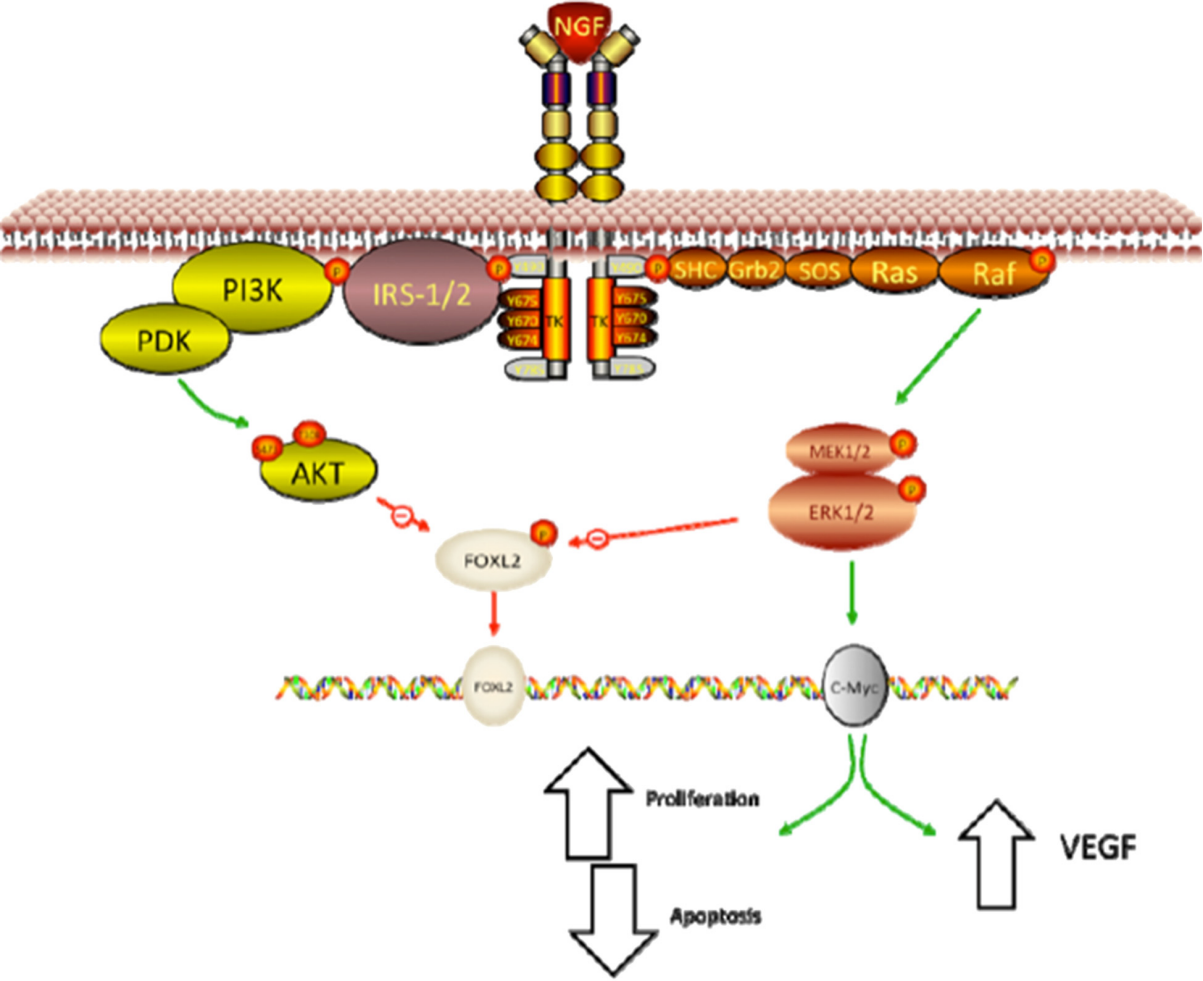
**Signaling pathways of TRKA activation by NGF.** The NGF-dependent activation pathway of TRKA is involved in cellular proliferation, survival, differentiation and angiogenesis in EOC.

In addition, a microarray study was performed to evaluate changes in some genes involved in the previously mentioned signaling pathway in EOC explants, and to compare them with a normal human ovarian epithelial surface cell line (HOSE). Such results evidenced an important increase of TRKA gene among EOC as compared to HOSE cells and also an increase in MAPK1, PI3K, and AKT2, without changes in FOXL2 gene [4] (Figure 1).

### Relationship between NGF, TRKA and VEGF in EOC angiogenesis

Autocrine and paracrine production of VEGF is the critical component of angiogenesis both in normal as well as in pathological tissues.

As a product of alternative splicing of the gene, five VEGF isoforms have been detected in mammals: VEGF121, VEGF145, VEGF165, VEGF189 and VEGF206 transcripts [47]. Transcripts coding for VEGF121, VEGF165 and VEGF189 are detected in the majority of cells expressing the VEGF gene, in which the main molecular species is VEGF165. On the contrary, VEGF206 is a rare form and has only been described in human fetal liver [20,48]. VEGF189 and VEGF206 have been observed to bind to heparin and to be almost completely sequestered in the extracellular matrix. On the other hand, an important fraction of VEGF121, VEGF145 and VEGF165 are secreted: however, an important fraction of VEGF165 remains bound to the cell surface and the extracellular matrix [49].

The predominant expression of VEGF121 and VEGF165 isoforms mRNA has been demonstrated in normal ovary and EOC [49,50]. The mRNA for such isoforms are overexpressed in most human tumors, including ovarian tumors [50]. When correlating VEGF expression with tumor characteristics, increased levels of VEGF165 have been found in all ovarian carcinoma stages, regardless of their histological type [50]. A study carried out on ovarian carcinoma cell lines and ovarian tumors in different stages demonstrated a higher predominant expression of VEGF121 and VEGF165; however, the VEGF189 isoform was found in two of six of the studied cell lines, and in some cases of ovarian cancer in more advanced stages [51].

The results obtained by our group show that mRNA levels of the three VEGF isoforms (VEGF121, VEGF165 and VEGF189) are found both in normal ovarian tissue as well as in EOC [8]. When comparing such isoform transcripts expression in both tissues there is a significant increase of VEGF165 and VEGF189 transcripts in EOC [8]. Moreover, when studying NGF – induced VEGF expression in cultured EOC explants; we found that NGF generates a significant increase of both mRNA as well as of the protein of the three above-mentioned isoforms [8]. Such effect was inhibited by NGF immuno-blockade and by a tyrosine kinase inhibitor (K252a), thus indicating that VEGF expression regulation by NGF in EOC is specific and mediated by the activation of its TRKA receptor [8].

There is practically no information regarding TRKA receptor expression in normal OSE cells. However, there are two studies [12,52] conducted with a small number of samples, where expression of such receptor in that epithelium could not be found. By virtue of detecting NGF and TRKA with immunohistochemistry in a larger number of normal ovary samples, our group found a mild NGF detection and even lower TRKA immunodetection in OSE in 10% of the cases studied [8]. Additionally we have also found NGF and TRKA expression in EOC tissues, similar to literature reports [46,52]. Interestingly, upon determination of mRNA and protein levels for NGF, TRKA and VEGF in normal ovary, benign tumors, borderline tumors, well differentiated EOC, moderately differentiated ovarian cancer and poorly differentiated ovarian cancer specimens, we found a sustained increase of NGF, TRKA and VEGF both transcripts as well as proteins in the epithelia of such tissues, evidencing a correlation with EOC progression. However, the most important changes were found for the phosphorylated TRKA receptor (p-TRKA), proving that such receptor is chiefly activated as tissue differentiation is lost and thus, when tissue is more aggressive [53]. The latter suggests that p-TRKA might be a marker for poor prognosis. Furthermore, NGF, total TRKA total and p-TRKA immunodetection was positive in endothelial cells of the same tissues and with similar increases to those found in the epithelia of such tissues [53]. For such reason, we decided to evaluate the effect of NGF- and VEGF- conditioned media secreted by EOC explants and by an EOC cell line (A2780) on cell proliferation, migration and differentiation in a human endothelial cell line (Eahy926). A significant increase in endothelial cell proliferation, migration and differentiation (vasculogenesis) was observed. Such effect decreased when using a tyrosine kinase receptor inhibitor (A252a) and when immune-blocking NGF with an antibody [53].

## Conclusion

Neurotrophins were first discovered in the brain, and their signaling pathways have mostly been studied in neurons. However, NGF has several functions outside the nervous system, including important roles in the development and normal functioning of the ovary. Besides, the TRKA signaling pathway due to NGF stimulation has been linked to cancer before. A deregulation in NGF signaling has been shown to promote proliferation, migration, angiogenesis and metastasis in different cancers, including breast cancer, melanoma and pancreatic cancer.

**Figure 2:**
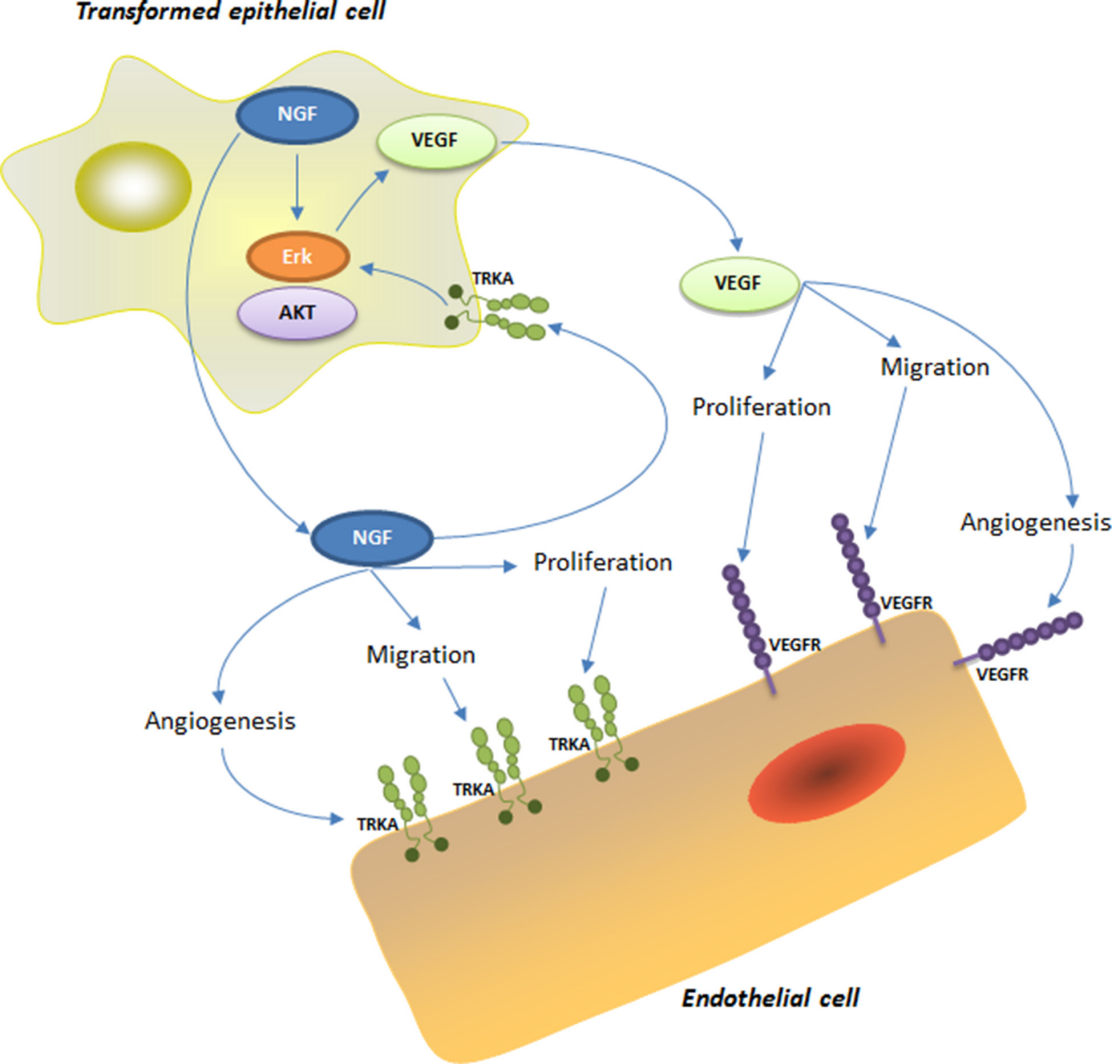
**NGF and its TRKA receptor are involved in the progression of EOC.** In EOC, NGF and its TRKA receptor are expressed in both epithelial and endothelial cells and are involved in important process such as angiogenesis during the tumor progression.

The data summarized above demonstrates that when NGF activates its high affinity receptor TRKA in human granulosa cells of normal ovary and in epithelial cells from EOC, it acts as an indirect angiogenic factor by increasing VEGF expression and also as a direct angiogenic factor by activating TRKA in endothelial cells, therefore increasing angiogenesis in EOC (Figure 2). Additionally, the above-described results enable us to conjecture that both NGF as well as TRKA receptor expression in normal OSE might be early events in EOC tumorigenesis and angiogenesis, giving us a better understanding in the function of neurotrophins in human carcinoma.

In the future, studies should focus on NGF levels on ovarian cancer patients, and its correlation with prognosis. Also, TRKA, the active TRKA form and NGF levels should be contrasted with survival after the current available therapies. Targeting the NGF-TRKA pathway could offer new approaches to the therapy of ovarian cancer, and it could be complementary to therapies that target VEGF. Also, further study is needed to elucidate if the relationship between angiogenesis, NGF, TRKA and VEGF is also present in other types of cancer.

## Abbreviations

Ang: Angiopoietin
BAX: BCL2-associated X protein
BCL2: B-Cell CLL/Lymphoma 2
BDNF: Brain-derived neurotrophic factor
EOC: Epithelial ovarian cancer
ERK: Extracellular signal-regulated kinase
FGF: Fibroblast growth factor
FGFR: Fibroblast growth factor receptor
FOXL2: Forkhead box L2
FSH: Follicular stimulating hormone
HOSE: Human ovarian surface epithelium
LH: Luteinizing hormone
MAPK: Mitogen-activated protein kinase
NGF: Nerve growth factor
OSE: Ovarian surface epithelium
PDGF: Platelet-derived growth factor
PDGFR: Platelet-derived growth factor receptor
PGE2: E2 prostaglandins
PI3K: Phosphatidylinositol 3-kinase
TRKA: Tyrosine receptor kinase A
VEGF: Vascular endothelial growth factor
